# Children and adults successfully comprehend subject-*only* sentences online

**DOI:** 10.1371/journal.pone.0209670

**Published:** 2019-01-17

**Authors:** Pooja Paul, Jayden Ziegler, Elizabeth Chalmers, Jesse Snedeker

**Affiliations:** 1 Department of Linguistics, Harvard University, Cambridge, MA, United States of America; 2 Department of Psychology, Harvard University, Cambridge, MA, United States of America; University of Haifa Faculty of Education, ISRAEL

## Abstract

In many offline studies, children show selectively better comprehension of sentences with the focus particle *only* when it modifies the object argument (*Jane only ate* *an apple*) than they do when it modifies the subject argument (*Only* *Jane* *ate an apple*). Here we explore the nature of this asymmetry by examining performance in a different kind of task: the moment-to-moment comprehension of unambiguous sentences. If past errors reflect a fundamental difference in representation or complexity of computation, we would expect the same asymmetry in this task. We observed that adults were able to successfully predict the target referent for both types of *only*-sentences, as indicated by anticipatory looks, while 6- to 8-year-old children could do so only for subject-modifying *only*-sentences. These findings suggest that much of the asymmetry in past work may be due to task demands. We discuss the implications of these results for children’s syntactic and pragmatic development.

## Introduction

To understand and use language, children must learn the meanings of words, as well as acquire the rules that allow them to construct more complex meanings from the basic parts. Some rules of composition merely combine forms (*red cup*), while others involve the assignment of participant roles (*The cat scared the dog*). In such cases, there appears to be a straightforward mapping between elements of the meaning (*redness*, *cats*, and *dogs*) and the words in the sentence.

Other compositional rules involve a more complex mapping of meaning to form. In a sentence like *Only Jane ate an apple*, there is no one-to-one mapping between an element in the event and the meaning of *only*. Instead, this focus particle encodes a particular logical relationship between the focused constituent within the sentence (*Jane*) and its contextually relevant alternatives (e.g., *Bob*, *Peter*, *Sue*), such that the sentence is true if and only if *Jane ate an apple* and *no one else* (*in the context*) *ate any apples*. Changing the position of *only* within the sentence changes what constituent it can associate with. For instance, if *only* is placed in pre-verbal position with focal stress on the object noun phrase (*Jane only ate* *an apple*), the meaning of the sentence changes dramatically. In this case, the domain of focus must involve alternatives to apples (e.g., *oranges*, *bananas*) rather than alternatives to *Jane*, and the corresponding sentence-level meaning becomes *Jane ate an apple* and *nothing else* (*in the context*). As such, understanding a sentence containing *only* requires integrating syntactic, lexical, and contextually-given information in order to arrive at the correct interpretation. The present study builds on prior work to address two linked questions: How does this integration happen in real time and how do these abilities develop in young children?

Previous developmental work on *only* has focused on children’s interpretation of *only*-sentences in offline comprehension tasks (see below), and typically found that they had greater success at comprehending sentences with pre-verbal *only* (object-*only*) compared with pre-subject *only* (subject-*only*). There has been an enduring puzzle about the possible source of this asymmetry, and what it reflects about children’s linguistic processing capacities [1–5]. One possibility is that it reflects fundamental differences in the representations and/or computations involved for the two sentence types. Another possibility is that the apparent asymmetry was simply a byproduct of the tasks involved. The present study aims to get to the bottom of this by asking whether children (and adults) are able to use the syntactic position of *only* as a cue to recover the underlying focus structure of the different *only*-sentences. We minimize task and memory demands compared to previous studies by providing a highly scaffolded and maximally coherent discourse context. Critically, we also use an implicit online measure (eye tracking) to gain insight into the time course of processing without imposing additional demands on the child. To succeed in our task, however, children must not only integrate the relevant syntactically-encoded information (position of *only*) with lexically-encoded content and contextual information, but they also must do so rapidly enough to make the appropriate predictions online. This study is, to the best of our knowledge, the first to investigate children’s *incremental* processing of *only* in different syntactic positions (though cf. [6] who investigated children’s incremental processing of *only*-sentences in Mandarin Chinese with different prosodic rather than syntactic cues). It is also the first eye-tracking study to show successful incremental processing of both subject-*only* and object-*only* sentences in English-speaking adults, contrary to previous findings.

In the remainder of this introduction, we present an informal, theory-neutral account of the meaning of the focus particle *only*. We then discuss past work on both children’s and adults’ comprehension of *only*, which provides theoretical ground for the present study.

### The meaning of *only*

The focus particle *only* has a complex meaning, which encodes a particular logical relationship between an explicitly stated proposition—i.e., the ‘prejacent’ (the sentence without *only*)—and an implicit domain of propositional alternatives to the prejacent. Consider the example in (1), a subject-*only* sentence in which *only* occurs sentence-initially and therefore modifies the subject.

(1) *Only Jane ate an apple*.

The sentence in (1) conveys that *Jane ate an apple* and that *no one else ate any apples*. Here, *Jane ate an apple* is the prejacent, and *no one else ate any apples* summarizes over a set of alternative propositions. But what, exactly, are these alternative propositions? Intuitively, *no one else ate any apples* cannot mean, literally, that no one else in the world and across all time besides Jane ate an apple, so this domain must somehow be restricted to a *contextually relevant* subset. This set of alternatives is usually constrained by the discourse context [7]. For example, suppose that we had already been talking about *Jane*, *Bob*, and *Susan* in the preceding discourse. Then the salient alternatives to *Jane* in that particular context would be *Bob* and *Susan*. Likewise, the relevant alternative propositions to the prejacent, *Jane ate an apple*, would be *Bob ate an apple* and *Susan ate an apple* (which *only* serves to ultimately negate). If we were instead discussing *Jane*, the *Pope*, and *George Bush*, then *Bob* and *Susan* cease to be salient alternatives to *Jane*. In this case, the relevant alternative propositions to the prejacent are now *The Pope ate an apple* and *George Bush ate an apple*. Thus, the set of contextually relevant alternatives for a given sentence containing *only* usually varies from one discourse context to another.

However, even keeping the discourse context constant, changing the syntactic position of *only* alone also radically changes the implicit domain of propositional alternatives. Consider the object-*only* sentence in (2), where *only* now modifies the object argument rather than the subject. The prejacent, or the explicitly-stated proposition, remains the same as before: *Jane ate an apple*. However, the relevant set of alternatives in (2) no longer involves contextual counterparts to *Jane*, but rather counterparts to the object argument, *an apple*.

(2) *Jane only ate an apple*.

Accordingly, the alternative propositions to (2) are now *Jane ate a pear*, *Jane ate an orange*, etc., on the basis of whatever alternatives to *apple* are salient in the discourse context, and *only* functions to negate the truth of just those propositions included in this domain.

In sum, the ability to correctly interpret sentences containing *only* requires listeners to integrate compositionally-derived meaning with contextually available material. Making predictions about upcoming discourse referents while listening to *only*-sentences further entails that these different sources of information from distinct cognitive systems be rapidly integrated in real time. Under many theories, context-sensitive processes do not come into play until a post-compositional stage (e.g., [8]). Our results therefore stand to bear directly on the empirical predictions of such theories.

### Past work on the comprehension of *only*

Previous experimental work on the comprehension of *only* has primarily fallen into two categories. On the one hand, we have studies of the acquisition of *only*. These studies have almost exclusively used offline measures of comprehension, like sentence-picture verification and the truth value judgment task, to test children’s ultimate interpretation of sentences containing *only*. On the other hand, some more recent work has also looked at how the position of *only* in the sentence can be used online as a cue during incremental processing. By taking into account the syntactic position of *only* in real time, listeners can identify the set of alternatives in the discourse as the sentence unfolds. This then allows them to narrow down the number of potential referents, differentially for subject- vs. object-*only* sentences, and make predictions about upcoming sentence content. The studies to date that have used online measures like eye tracking to examine the processing of *only-*sentences as they unfold in real time have primarily looked at adult participants. In the present study, we investigate 6- to 8-year-old English-speaking children’s capacity to integrate linguistic and pragmatic information in *only*-sentences online in order to gain a better understanding of the observed asymmetry in their offline performance. This age range was chosen because 3- to 6-year-old children in previous studies consistently show little to no ability to correctly interpret subject-*only* sentences offline (e.g., [9–11])), while work with children up to age 10 shows better, albeit still asymmetrical, performance (e.g., [2]).

#### Offline acquisition work

The offline comprehension work has been mixed with regard to whether and how well children understand sentences containing *only*. In some cases, children have been shown to comprehend object-*only* sentences in an adult-like manner while interpreting subject-*only* sentences in a non-adult-like manner [1, 9–15]. Crain and colleagues [1, 9, 15] have suggested that this asymmetry is due to syntactic mis-parsing, such that children incorrectly assign the scope of *only* in subject-*only* sentences to the verb phrase rather than to the subject, placing focus on the former but not the latter. However, subsequent research does not support this possibility. For example, Müller et al. [16] found the same asymmetry in subject-final German sentences in which *only* unambiguously takes scope over the subject and nothing else (3). Since these scopally unambiguous sentences cannot be mis-parsed, this finding suggests that the observed asymmetry is due to non-syntactic factors.

(3) *Den Ballon hat nur die Maus* the-M-ACC balloon-ACC has only the-F-NOM mouse-NOM ‘Only the mouse has the balloon’

Paterson and colleagues [2, 3] have suggested, instead, that children’s poor performance with *only*-sentences reflects their failure to mentally construct and represent the relevant sets of alternatives associated with *only*-sentences, especially when these sets are not made salient in the prior discourse but instead have to be inferred indirectly. This predicts, accordingly, improved comprehension when the set of alternatives is restricted either via the pictures used at test themselves [3] or when the alternatives are verbally introduced prior to test [11, 17] (see also [18] for object-*only* sentences alone). It turns out that under such circumstances children have better success with comprehending both types of *only*-sentences, albeit less well than adults. These results further undermine Crain and colleagues’ syntactic mis-parsing account. Nevertheless, even when children show above-chance comprehension with both types of *only*-sentences, they still seem to do better overall on object- vs. subject-*only* sentences [2, 5, 11, 19]. Thus, a general difficulty with pragmatically restricting the domain of *only* cannot account for the full range of data to date. Indeed, there may still be a fundamental, if non-categorical, difference in the processing of these two sentence types.

We consider two broad classes of possibilities. One hypothesis is that the asymmetry is due to a difficulty in integrating the syntactic and contextual information required to understand sentences with *only*. Making an offline judgment in one of these tasks requires children to first integrate the multiple sources of information required to determine the truth conditions of the sentence, and then to compare the information given in the task (usually a picture or set of pictures) against these truth conditions to arrive at an explicit final judgment as to whether the sentence is true or false. This process may be difficult for children, which would explain their poorer performance on *both* types of sentences relative to adult performance. However, Müller and colleagues [4, 11, 16] (see also [5] for a similar proposal) have further suggested that this process may be even more difficult for subject-*only* sentences, because though the default position for focus is typically on the sentence object (e.g., [20]), the syntactic position of *only* in a subject-*only* sentence requires that focus be assigned to the subject of the sentence instead. As a result, successfully processing a subject-*only* sentence requires successfully overriding the information structural default to correctly assign focus. Note that this is not an issue with object-*only* sentences, which are consistent with the default focus assignment. On this type of account, younger children, who generally have lower executive function abilities (for a review, see [21]), may prioritize certain types of information—in this case, the information structural default—and therefore be less able to override a reading of the sentence that conflicts with it. This would lead to slower resolution of this conflict during comprehension and, as a result, decreased performance on subject-*only* sentences in comparison to object-*only* sentences (for further discussion, see [4]). We’ll refer to this possibility as the *processing difficulty hypothesis*.

An alternative hypothesis is that the performance asymmetry observed in children for subject- vs. object-*only* could instead be due to difficulties inherent to the tasks used to measure the offline comprehension of these types of sentences. For instance, children may struggle with the metalinguistic component of sentence-picture verification and truth value judgment tasks, despite having comprehended the sentences just fine. In these experiments, children are shown a display consisting of the target character and two to three distractor characters making up the contrast set, with each character holding one to two items (e.g., a flag, a ball, etc.). Thus, in order to make the object-*only* inference, the child must simply locate the target character and compare the small number of items it is holding to the truth conditions of the sentence. To make the subject-*only* inference, however, the child must instead compare each of the distractor characters’ object sets to the sentence’s truth conditions, effectively negating across a larger and more complicated set of alternatives. The more complicated verification strategy required for subject-*only* sentences in such tasks, coupled with children’s lower metalinguistic abilities in general (see, e.g., [22]), may well explain the observed asymmetry in children’s performance with sentences containing the focus particle *only*, rather than being diagnostic of a deeper difference in kind between the two types of *only*-sentences. We will refer to this as the *task difficulty hypothesis*. Some researchers have suggested that subject-*only* behaves like a determiner, which unlike other determiners, is non-conservative (e.g., *Only A are A that B). This could make it harder to verify, explaining the asymmetry in the acquisition work. However, there are at least two reasons to doubt this analysis. First, *only* in subject position can associate with full DPs, which other determiners cannot do (e.g., *Only the children had lemonade* vs. *Those the children had lemonade). In addition, conservativity is a defining property of determiners; since subject-*only* is non-conservative, it is not likely to be a determiner. We therefore refrain from discussing this possibility further.

Given that adults’ accuracy rates are at ceiling on offline versions of these tasks [5, 23], one conclusion we could draw from the acquisition work thus far is that the offline asymmetry is specific to children. Under the *processing difficulty hypothesis* outlined above, this results from children’s inability to resolve the conflict between the focus structure of subject-*only* sentences and the information structural default to arrive at a final interpretation of the sentence, whereas adults do so successfully. Importantly, this hypothesis would predict children’s performance in *online* tasks to be equally asymmetrical. Under the *task difficulty hypothesis*, on the other hand, the reported asymmetry merely reflects the differential complexity of the verification strategies required for the tasks used in previous work. Accordingly, this latter hypothesis predicts children’s online processing of subject-*only* sentences to be no more challenging than that of analogous object-*only* sentences, so long as task demands are minimized and comparable for both sentence types. We might therefore expect to see adults as well as children successfully predicting upcoming discourse referents for both types of *only*-sentences online. To gain traction on these two hypotheses, we now turn to a review of the online processing work, primarily among adults, but which includes a single eye-tracking study in children as well.

#### Online processing work in adults

Kim, Gunlogson, Tanenhaus, and Runner [24,25] investigated the online comprehension of object-*only* sentences in adults using eye tracking in the visual world. In this study, participants were presented with a discourse consisting of a sentence introducing a background character (*Neil*) and two items that the character had (*apples* and *cards*). This was followed by a second, critical sentence introducing a target character (*Jane*) and the item that this character had (*apples*). This critical sentence was either an object-*only* sentence (*Jane only has some apples*) or a control sentence without *only* (*Jane has some apples*). Participants listened to these discourses while looking at a visual display consisting of the target item (*apples*), a competitor item with the same phonological onset (***a****xes*), and two distractor items (*skates*, *medals*).

What Kim et al. [25] observed was that participants reliably looked predictively to the target item for object-*only* sentences, but only when it was explicitly mentioned in the prior discourse, an effect we’ll refer to as the *previous mention bias*. Specifically, participants considered as expected members of the contrast set just those items that had been verbally introduced in the sentence preceding the critical sentence (*Neil has some apples and some cards*), rather than all possible referents in the visual context (*apples*, *axes*, *skates*, and *medals*). Thus, upon hearing the onset of the target word in the critical sentence (*Jane only has some* ***a*** …), which was consistent with two items in the display that are logically congruent with the semantics of object-*only* (*apples*, *axes*), participants looked reliably more to the target when it was a member of the set of items *Neil* had (in this case, *apples*), as guided by the previous mention bias, but equally to *apples* and *axes* when it was not (see also [26, 27, 28]).

Similarly, an eye-tracking study by Romoli, Khan, Sudo, and Snedeker [23] examined adults’ incremental processing of subject-*only* sentences in comparison with another focus-sensitive particle, *also*. The discourses in Romoli et al. [23] (Exp. 2) had the same general structure as those in Kim et al. [25], including an introductory sentence highlighting the items that a background character had, either explicitly or implicitly (*Michael has got candies and watches* vs. *Look at what Michael has*!), and a following critical sentence either containing subject-*only* (*Only Sarah has some candles*) or not (*Sarah has got some candles*). However, rather than displaying the object items individually, as in Kim et al. [25], Romoli et al.’s [23] (Exp. 2) displays paired sets of items with characters. The character image at the top of the display uniquely matched the gender of the background character (*Michael*), and appeared next to images of the two objects mentioned in the context sentence (*candies*, *watches*). Appearing in the bottom half of the display were two potential referents for the character mentioned in the critical sentence (*Sarah*), one paired with an item familiar from the preceding context (***cand****ies*) and the other paired with a novel item sharing a phonological onset with the familiar item (***cand****les*).

Crucially, given the truth conditions of a subject-*only* sentence within the specified context, the only semantically congruent referent for *Sarah* would have to be the gender-matched character with the novel/unique item (*candles*). The authors reasoned that if adults quickly integrated this semantic constraint imposed by the meaning of *only* when it modifies the subject incrementally (i.e., as the sentence unfolded), they would see anticipatory looks to the character appearing with the novel (i.e., unmentioned) member of the phonological cohort pair. Specifically, at the onset of the target word (***cand*** …), participants should look more to the gender-matched character with *candles* (novel) rather than to the one with *candies* (mentioned). However, Romoli et al. [23] failed to find such an online novelty preference in their sample of adults. Another experiment in the same paper [23] (Exp. 1) used a similar structure to Kim et al. [25], with the target displays containing four items rather than pairings of items with characters. However, the contextual setup in this experiment was less natural, making interpretation of the null result for subject-*only* less clear (for further discussion, see [23]). A follow-up study by Paul et al. [26] directly comparing the online processing of subject- vs. object-*only* sentences in adults once again found this failure with subject-*only* sentences, despite successfully replicating Kim et al.’s [25] previous mention bias with object-*only* sentences.

One interpretation of this pattern of results is that subject-*only* sentences may be more computationally taxing than object-*only* sentences even for adults, hindering predictive processing despite ceiling performance in offline measures. This interpretation is consistent with the *processing difficulty hypothesis*. On this hypothesis, using subject-*only* to make an inference about the correct target noun requires the additional step of overriding the default focus assignment to correctly assign focus before the appropriate alternatives can be properly identified and negated. This step incurs an additional processing cost that slows down adult listeners enough to prevent them from predicting the target object within the brief ambiguous period at the onset of the critical noun. They ultimately complete the calculation in spite of the slowdown, resulting in ceiling performance on offline measures.

Critically, the adult pattern of findings is also consistent with the *task difficulty hypothesis*. That is, it is not clear that adults’ difficulties with online target prediction in subject-*only* sentences reflects additional complexity inherent to subject-*only* sentences per se, rather than being a consequence of the particular tasks used in the recent online studies. Computing the object-*only* inference requires negating over objects associated with a single individual (e.g., the items that *Jane* has/doesn’t have) to arrive at the correct interpretation, while computing the subject-*only* inference requires negating over a *set* of individuals (subject-alternatives) and their corresponding objects. Thus, when there are multiple individuals/scenes in the contrast set against which the truth conditions of subject-*only* sentences must be verified, as in both Romoli et al. [23] (Exp. 2) and Paul et al. [26], the subject-*only* inference is quite complicated because each potential referent must be ruled out individually. In contrast, computing the object-*only* inference might simply require identifying the candidate referent(s) with just one item in their possession, and a robust previous mention bias on top of that driving looks to the character with the already-mentioned item. This latter inferential process is arguably less costly to compute, in part as a consequence of the task design.

#### Online processing work in children

Recent results in children by Höhle et al. [4] for the German counterpart of *only* (‘nur’) seem, at least at first glance, to favor the *task difficulty hypothesis*. In a visual-world eye-tracking study of German speaking children, Höhle et al. [4] found that 4-year-olds displayed reliably different eye gaze patterns for their subject-‘nur’ (subject-*only*) vs. object-‘nur’ (object-*only*) conditions. Children were presented with a visual display containing a target character and three distractor characters (i.e., the contrast set), which they passively viewed as they listened to a sentence containing either a pre-subject ‘nur’ (*only*) or a pre-object ‘nur’ (*only*). The authors reasoned that in order to verify whether a subject-‘nur’ (subject-*only*) sentence matches the scene, one must visually interrogate the contextual alternatives to the subject; this is not the case for object-*only* sentences. They found that, like their adult controls, children indeed looked more to the three distractor characters (subject-alternatives) after hearing subject-*only* sentences compared to after hearing object-*only* sentences. Thus, with a simpler (implicit) measure, 4-year-old children appear to correctly identify the quantificational domain of subject-*only* sentences, despite chance performance by the same children in their offline behavioral responses.

However, these results have important limitations. First, the reported effects in looking behavior occurred 1.5 to 4.5 seconds *after* the sentence offset, rather than during the sentence, so they are at best indirect evidence for children’s real-time comprehension. More importantly, these findings have a simpler alternative explanation. In Höhle et al.’s [4] materials, half of the time the critical sentence was true in the context of the visual display, while the other half of the time the sentence was false. In the subject-*only*/True cases, the characters in the contrast set had items that differed from that of the target character’s (e.g., the target character had a kite and the other three characters had balloons). In the subject-*only*/False cases, in contrast, the other characters had the *same* item as the target character (e.g., all four characters had kites). Crucially, Höhle et al. [4] found the different looking pattern for subject-*only* sentences *exclusively* when the sentence was false given the visual display (i.e., all four characters had a kite). Thus, children looked more to the three other kites when they heard the word *kite* in the condition in which all four characters had kites, but they did not look more to the three balloons when they heard the word *kite* in the condition in which just the target character had a kite. These results can thus more simply be summarized as: People tend to look at the referents of things that are mentioned. Accordingly, although suggestive, this study leaves open whether children can indeed interpret subject-*only* sentences correctly in an online language comprehension task.

#### Summary of past work on *only*

In sum, there are no clear data to date showing children’s successful comprehension of subject-*only* sentences using online measures of comprehension. Nevertheless, offline comprehension data show that children tend to perform worse on subject-*only* sentences than object-*only* sentences, even when they show above-chance success with both (e.g., [2, 11, 19]). This suggests that children *do* have the requisite representations to interpret sentences with both subject- and object-*only* as part of their grammar. However, these findings from offline measures represent only part of the empirical picture. Online and offline measures ultimately show two different facets of language comprehension (moment-to-moment understanding as the sentence unfolds vs. ultimate interpretation of sentence meaning after the sentence ends). The lack of clear online data to date therefore leaves us with an incomplete picture of children’s processing of sentences containing *only*. We must complete this picture in order to understand the exact nature of the difficulty children face in making use of and integrating the representations involved in the comprehension of subject-*only* sentences in particular.

### The current study

The present study aims to provide a more complete picture of how children interpret sentences with *only* on a moment-to-moment basis. To this end, we adopted the eye-tracking procedure used in the adult literature [23, 25, 26] because this allows us to examine children’s understanding of the sentence as it unfolds in real time. Since the effects observed in the adult processing literature have been found at the onset of the target noun (see [23, 25, 26]), the increased temporal sensitivity of this measure is therefore necessary to determine whether children can process subject-*only* sentences online as rapidly as they process object-*only* sentences, and whether the pattern of results they exhibit is similar to that in adults.

We simplified our task considerably from Paul et al. [26] to address our concerns about the influence of task-specific demands on previous findings, and also to make it more suitable for children. In an effort to reduce the complexity of the task, we limited the number of possible referents to just two items sharing a phonological onset (target, distractor). Some past work on the online processing of *only*-sentences in adults [23, 26] has used materials that include multiple characters of the same gender in the visual display, each possessing one to two items. On the basis of the unfolding *only*-sentence, the task is to identify which of the characters in the display possesses the appropriate item(s) and is therefore the target. Still other work has fixed the intended character but included four possible reference items [23, 25]. By limiting our materials to include just two potential referents for the target item, we further reduce the space of possibilities to consider, and thereby simplify the calculation of the target inferences. Moreover, we also move to a blocked rather than interspersed design to further decrease task demands for children in particular (see [29]). Accordingly, if children’s and adults’ apparent difficulty with the offline and online comprehension, respectively, of subject-*only* sentences in prior work is attributable to methodological factors or task demands alone, we might expect them more likely to succeed on our simplified task. If the difficulty instead stems from children’s trouble overriding the default focus structure in the case of subject-*only*, then we should see the same asymmetry in performance as before.

Before we can draw more general conclusions from our child findings, however, we must first establish whether the online processing asymmetry in adults indeed goes away in our more simplified design. If not, then perhaps the asymmetry reported in the acquisition literature is in fact due to a genuine difference between subject- and object-*only* sentences rather than to task demands. To this end, in Experiment 1, we collect data from adults on our simplified paradigm and blocked design in order to verify that they can in fact successfully use subject-*only* as a cue during online processing when the complexity of the task is sufficiently reduced. Experiment 2 further asks whether the observed pattern of results in Exp. 1 extends to an interspersed rather than blocked design, to allow for more direct comparison with the findings in past work [26]. Having established the pattern in adults, we ask in Experiments 3 and 4 whether children can also successfully use subject-*only* as a cue during online processing in our more simplified design. These experiments were approved by Harvard University’s Institutional Review Board (Committee on the Use of Human Subjects), and written consent was obtained from all participants and/or their guardians prior to their participation.

## Experiment 1

### Methods

#### Participants

24 native English-speaking adults (17 female, 7 male; mean age = 23 [SD = 4], range = 18–31) recruited from Harvard University participated in the experiment. The participants received course credit for their participation.

#### Materials

**Fig 1:**
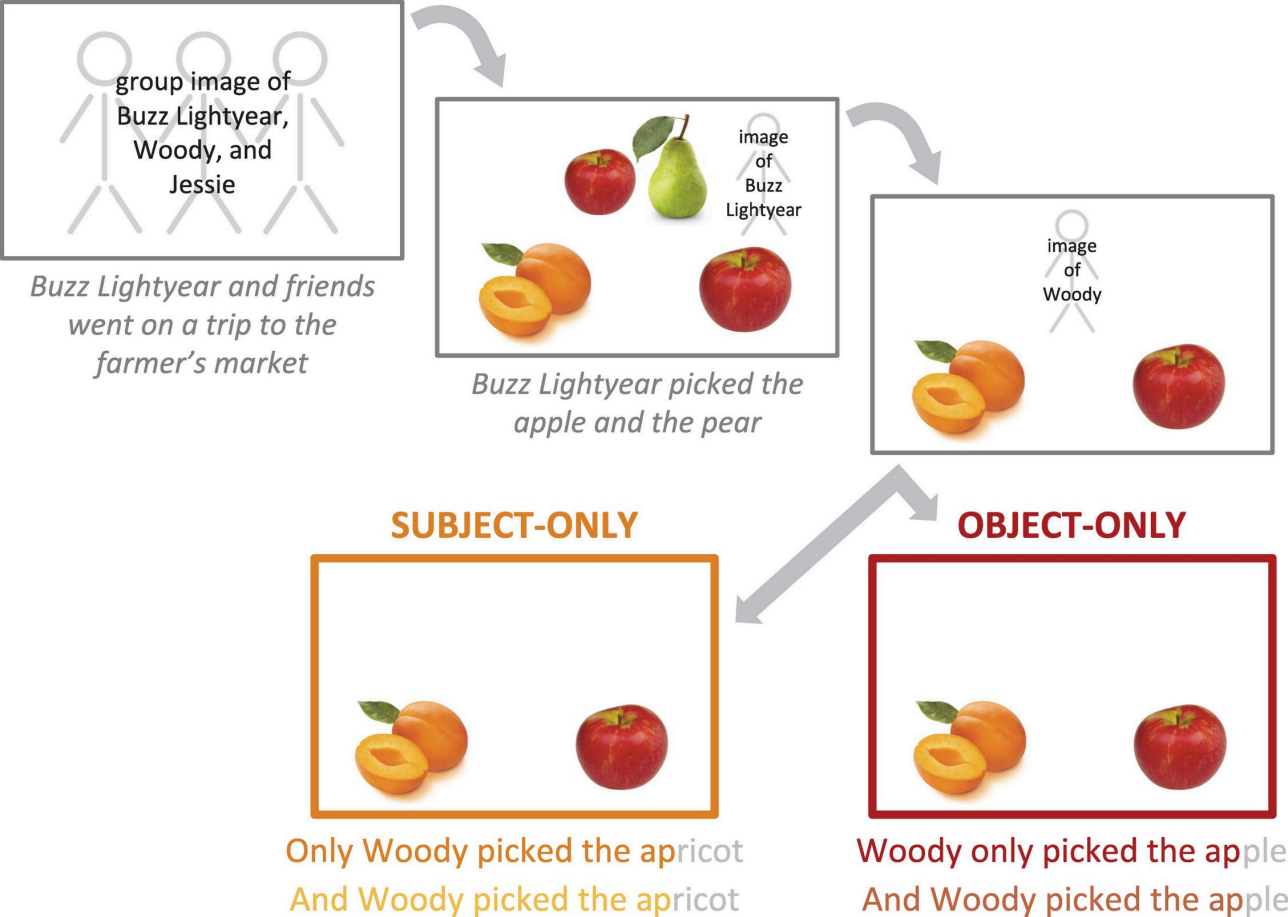
Procedure and example materials for all experiments.

The study consisted of 24 critical trials (12 Only, 12 Control) interspersed with 24 filler trials, for a total of 48 trials. Critical trials were blocked, such that participants encountered one block of Subject trials (6 Only, 6 Control) and a second block of Object trials (6 Only, 6 Control), or the reverse, with block order counterbalanced across participants. We used a blocked design to further decrease task demands and increase our chances of finding the intended effect, should it in fact exist. Each block contained 24 trials (12 critical, 12 fillers). All trials were embedded within a frame-tale about groups of friends going on different adventures together, and picking their “favorites” at the end of each adventure. Each trial included a verbal introduction to the characters involved (3), a description of what the background character picked as their favorites (4), and a critical sentence describing what the target character picked (5), each presented with a corresponding visual display (Fig 1).

(3) *Buzz Lightyear and friends went on a trip to the farmer’s market*.
(4) *Buzz Lightyear picked the apple and the pear*.
(5) a. ***Only** Woody picked the apricot.* = Subject-Only
      b. ***And** Woody picked the apricot*. = Subject-Control
      c. *Woody **only** picked the apple*. = Object-Only
      d. ***And** Woody picked the apple*. = Object-Control

During the introductory sentence to each vignette (3), three characters appeared together as a group in the visual display. This was done to familiarize participants with who the subsequent sentences were going to be about. Next, participants heard a description of what the first character picked (4) while viewing a visual display featuring an image of that character and the items s/he picked in the upper half of the display and two additional items in the bottom two quadrants of the display. One of these bottom two items corresponded to an item that the background character had picked (indicated visually using the exact same image), while the other was novel but shared a phonological onset with the previously mentioned item (since the previously mentioned item was identical to that of the target character’s, this licensed the use of the definite article *the* in the object-*only* sentences). Then, the background character and his/her items was replaced by an image of the target character, and the critical sentence was played (5). At the end of the critical sentence, the target character appeared next to the item chosen by him/her (location of the target item was randomized across trials and lists).

Critically, the final word in each critical sentence shared an onset with the label for the only other possible referent in the display (e.g., ***ap****ple/****ap****ricot*), leading to temporary ambiguity between the two potential referents. We’ll refer to this pair as a *phonological cohort*. (See S1 Table for full list of critical materials.) We were interested in whether participants would correctly anticipate the different intended referents of the two types of *only-*sentences based on information provided in part by the syntactic position of *only* (*apricot* for Subject, *apple* for Object), as measured by greater early looks to the target than the distractor during the brief period of ambiguity following the onset of the final noun, relative to the controls. All sentences were pre-recorded by a female adult native speaker of American English and presented to participants over speakers. Subject- and object-*only* sentences typically differ in prosodic structure, with relatively more stress (= longer duration) on the subject in subject-*only* sentences and relatively more stress on the object in object-*only* sentences. This additional cue to focus structure could lead to correct performance on our task independent of (or in addition to) the syntactic structure. To isolate syntax as the sole predictor of focus structure, we were careful in recording our sentences to match subject and object durations, respectively, across the two conditions. Two-sample *t*-tests revealed that our subject durations were equivalent across subject- and object-*only* sentences, *t*(92) = .60, *p* = .55, and so were our object durations, *t*(93) = .10, *p* = .92. Thus, the only reliable cue to focus structure in our experiments was the syntactic position of *only*.

Filler trials resembled control trials (with *and* instead of *only*) and had the same overall structure. Since all critical trials in the Subject block had the novel referent as the target, and all critical trials in the Object block had a previously mentioned referent as the target, fillers were constructed so as to counteract these strong tendencies, as well as to balance the total number of novel and previously mentioned targets across the entire experiment. Accordingly, for the Subject block, we included 10 filler items with a previously mentioned object as the target referent and 2 with the novel object as the target referent; for the Object block, we included 10 filler items with the novel object as the target referent and 2 with a previously mentioned object as the target referent. Thus, across the entire experiment, 24 target items were novel (12 critical items in Subject block, 2 filler items in Subject block, 10 filler items in Object block), and 24 target items were previously mentioned (12 critical items in Object block, 2 filler items in Object block, 10 filler items in Subject block). Of the filler items in a given block, exactly half included a phonological cohort, as in the critical items, and the other half did not. Because the Control items and filler items had ostensibly the same structure, these measures ensured that slightly over half of the trials containing *and* in a given block pushed in the opposite direction as those with *only* (10/18 previously mentioned targets in the Subject block, 10/18 novel targets in the Object block), while two-thirds of these items (12/18) contained a phonological cohort and one-third did not. Filler items were the same across lists.

#### Procedure

All experiments were administered in the lab using E-Prime (Psychology Software Tools, Pittsburgh, PA). On critical sentences, participants were asked to touch the picture that matched what the second character picked. Target displays remained on the screen until participants made their selection. All behavioral responses and eye gaze data were recorded by E-Prime. For adult participants, a post-test questionnaire confirmed that none of the participants realized the true purpose of the experiment.

#### Design

We used a 2 × 2 × 2 mixed design, with Condition (Only, Control) and Syntactic Position (Subject, Object) as within-subjects factors, and Block Number (1, 2) as a between-subjects factor. As our dependent variable, we used a binary measure indicating whether participants looked more at the target or distractor image during the 200-ms time window post-noun onset (corrected by 200 ms for saccade planning [30]) in which the identity of the intended referent was temporarily ambiguous. To calculate this measure, we averaged looks, separately, to both the target image and the distractor image in this time window for each trial. We then divided the proportion of looks to the target image by the total proportion of looks to the target and distractor images (T/T+D). If this value was greater than 0.5, we coded it as 1. If it was less than 0.5, we coded it as 0. If participants looked at both images equally, then we coded the trial as NA. Thus, we had one data point per item per participant. In presenting the results (for descriptive purposes), we have aggregated over both participants and items. Participants were randomly assigned to one of eight counterbalanced lists.

#### Data analysis

The eye-tracking data were time-locked to the onset of the target noun and averaged into 100-ms time bins spanning the entirety of each sentence. Individual trials were excluded from analysis if fewer than 30 frames (half a second) included valid eye gazes (e.g., participants mostly not looking at the screen, poor tracking, etc.), resulting in 6.6% trial loss.

The data for Exp. 1 were analyzed using a logistic mixed-effects model [31, 32] in the lme4 package in R [33], with Condition (Only, Control), Syntactic Position (Subject, Object), Block Number (1, 2), and their interactions as fixed effects. Maximal random effects structure [34] did not significantly improve model fit, *χ*^2^(18) = 10.53, *p* = .91, so only random intercepts for participant and item (vignette) were included in the final model. All fixed effects were effect coded. Follow-up comparisons used the same base model minus the relevant fixed effect.

### Results

**Fig 2:**
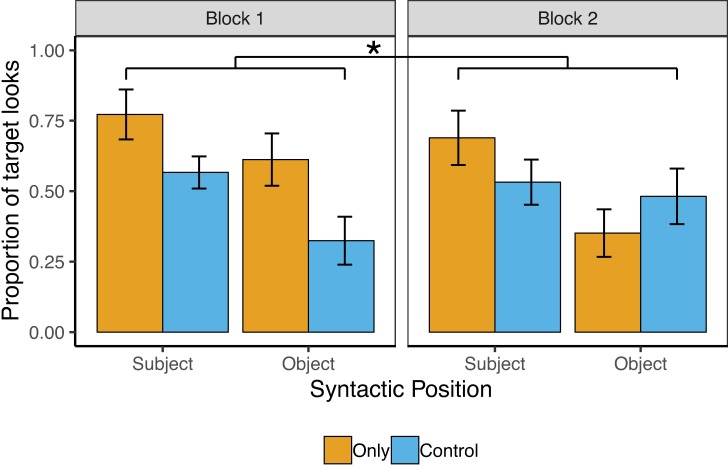
Adult results from Exp. 1. Error bars reflect by-subject standard errors.

Target accuracy on the behavioral task was perfect (100%), with no differences by condition. Fig 2 shows the pattern of results for Exp. 1. The overall model revealed significant main effects of Condition and Syntactic Position, such that adult participants looked significantly more to the target image in sentences with Only vs. their Controls (61% vs. 48%), *β* = .30(SE = .11), *z* = 2.71, *p* = .007, and significantly more to the target image in sentences in which *only* was in Subject vs. Object position (65% vs. 43%), *β* = .31(SE = .11), *z* = 2.76, *p* = .006, respectively. There was also a significant overall Condition-by-Block-Number interaction, *β* = .23(SE = .11), *z* = 2.07, *p* = .04, which follow-up analyses revealed was due to a significant increase in looks to the target image for Only over Control sentences in Block 1 (70% vs. 45%), *β* = .54(SE = .16), *z* = 3.35, *p* < .001, but not in Block 2 (51% vs. 51%), *β* = .08(SE = .16), *z* = .47, *p* = .64.

### Discussion

We find successful online prediction of upcoming discourse referents by adults for both subject-*only* sentences and object-*only* sentences, contrary to previous findings. Thus, by moving to a more simplified task, we have shown that adults are capable of successfully processing both types of *only*-sentences rapidly online. However, adults successfully used the presence and position of *only* for incremental prediction in the first block of our experiment, but not the second. This is compatible with at least two explanations.

First, adults may become less engaged in the study by the second block, due to boredom. This might seem at odds with the 100% accuracy we saw on the behavioral task. However, since the behavioral task requires only that participants pay attention to the last word in the sentence, it is possible that they may still be performing well on it despite not engaging with the fully composed meaning of the sentence itself.

Second, and perhaps more plausibly, adults may instead experience interference when required to switch from one sentence type to the other at the end of the first block. Given the blocked nature of this study, it’s impossible at present to tease these two possibilities apart. We address this concern in Exp. 2, in which we switch from a blocked design to an interspersed design. Doing so will also allow us to compare our results more directly to the past adult work. It is, in principle, possible that participants merely learned an experiment-specific rule—e.g., if subject-*only*, then unmentioned item; if object-*only*, then previously mentioned item. Evidence against this possibility is the fact that participants in our task showed the expected looking behavior already on the first critical trial in Block 1 (prior to any possible learning having taken place): Participants whose first critical trial was a Subject-Only sentence looked to the target 100% of the time during the ambiguous window, while participants whose first critical trial was a Subject-Control sentence only looked to the target 40% of the time, and similarly for Object-Only vs. Object-Control sentences (80% vs. 33%).

## Experiment 2

### Methods

#### Participants

24 native English-speaking adults (13 female, 11 male; mean age = 20 [SD = 2], range = 18–24) recruited from Harvard University participated in the experiment. The participants received course credit for their participation.

#### Materials

The materials for Exp. 2 were the same as for Exp. 1, with one notable exception: Rather than blocked, Subject and Object trials within each list were randomly interspersed, following Paul et al. [26].

#### Procedure

The procedure for Exp. 2 was the same as for Exp. 1. A post-test questionnaire confirmed that none of the participants realized the true purpose of the experiment.

#### Design

We used a 2 × 2 design, with Condition (Only, Control) and Syntactic Position (Subject, Object) as within-subjects factors. Our dependent variable was the same as in Exp. 1. Participants were randomly assigned to one of eight counterbalanced lists.

#### Data analysis

Data preparation for Exp. 2 was the same as for Exp. 1, resulting in 12.2% trial loss. We analyzed the data using a logistic mixed-effects model in the lme4 package in R, with Condition (Only, Control), Syntactic Position (Subject, Object), and their interaction as fixed effects. Maximal random effects structure did not significantly improve model fit, *χ*^2^(10) = 3.56, *p* = .97, so only random intercepts for participant and item (vignette) were included in the final model. Both fixed effects were effect coded. Follow-up comparisons used the same base model minus the relevant fixed effect.

### Results

**Fig 3:**
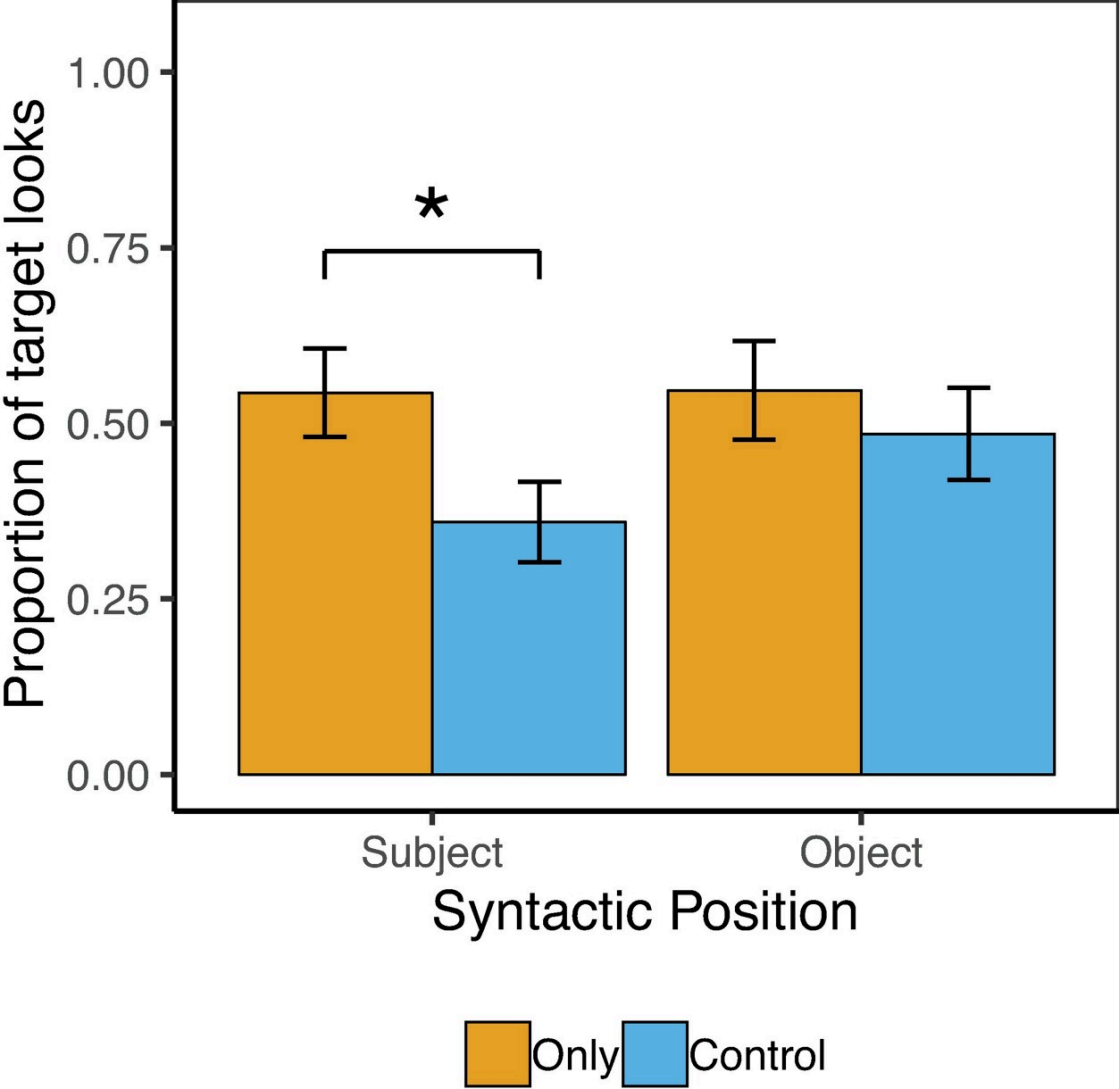
Adult results from Exp. 2. Error bars reflect by-subject standard errors.

Target accuracy on the behavioral task was perfect (100%), with no differences by condition. Fig 3 shows the pattern of results for Exp. 2. The overall model revealed a significant main effect of Condition, such that participants looked significantly more to the target during the ambiguous window in the Only conditions relative to the Control conditions (56% vs. 40%), *β* = .27(SE = .12), *z* = 2.29, *p* = .02, which follow-up analyses revealed was true for Subject-Only sentences (54% vs. 36%), *β* = .44(SE = .17), *z* = 2.54, *p* = .01, but not for Object-Only sentences (55% vs. 48%), *β* = .11(SE = .17), *z* = .67, *p* = .51. There were no other main effects or interactions.

### Discussion

As in Exp. 1, participants in Exp. 2 showed success overall in using *only*-sentences to make predictions about upcoming discourse referents. However, unlike in Exp. 1, this success appeared to be driven by subject-*only* sentences, while the contrast of object-*only* sentences vs. their controls did not reach statistical significance. Given that the pattern of responses to object-*only* sentences does in fact trend in the expected direction, this could potentially be due to our study having insufficient power to detect the difference (for a medium effect size, Cohen’s *d* = 0.5, we would have needed 34 participants for 80% power and 44 for 90% power). Nevertheless, these results make it clear that adults have difficulty interpreting both types of *only*-sentences to the same degree within a single experimental session, regardless of whether the sentences are presented in a blocked (Exp. 1) or interspersed (Exp. 2) design, consistent with past work (e.g., [26]). We return to this issue in the general discussion.

Importantly for our purposes, despite adults’ failure to show a previous mention bias with object-*only* sentences in Exp. 2, we did replicate the success with subject-*only* sentences from Exp. 1. Thus, our revised task appears overall to be well-suited to revealing successful performance for sentences containing subject-*only* in particular. In Exp. 3, we used the same blocked design from Exp. 1 in order to determine whether children are also able to successfully interpret subject-*only* sentences online under these circumstances.

## Experiment 3

### Methods

#### Participants

40 native English-speaking 6- to 8-year-old children (26 female, 14 male; mean age = 7;4 [SD = 11 mos.], range = 6;0–8;10) recruited from the greater Boston area participated in the experiment. The children received a toy for their participation, and their guardians received a $5 travel reimbursement.

#### Materials

The materials for Exp. 3 were the same as for the previous experiments.

#### Procedure

The procedure for Exp. 3 was the same as for the previous experiments.

#### Design

We used the same design as in Exp. 1, with one notable exception: For all child analyses, we used a 300-ms time window in calculating our dependent variable to give children more time to process the auditory information and launch a saccade (e.g., [35]).

#### Data analysis

Data preparation for Exp. 3 was the same as for the previous experiments, resulting in 5.7% trial loss. We analyzed the data using a logistic mixed-effects model in the lme4 package in R, with Condition (Only, Control), Syntactic Position (Subject, Object), Block Number (1, 2), and their interactions as fixed effects. Maximal random effects structure did not significantly improve model fit, *χ*^2^(7) = 4.09, *p* = .77, so only random intercepts for participant and item (vignette) were included in the final model. All fixed effects were effect coded. Follow-up comparisons used the same base model minus the relevant fixed effect.

### Results

**Fig 4:**
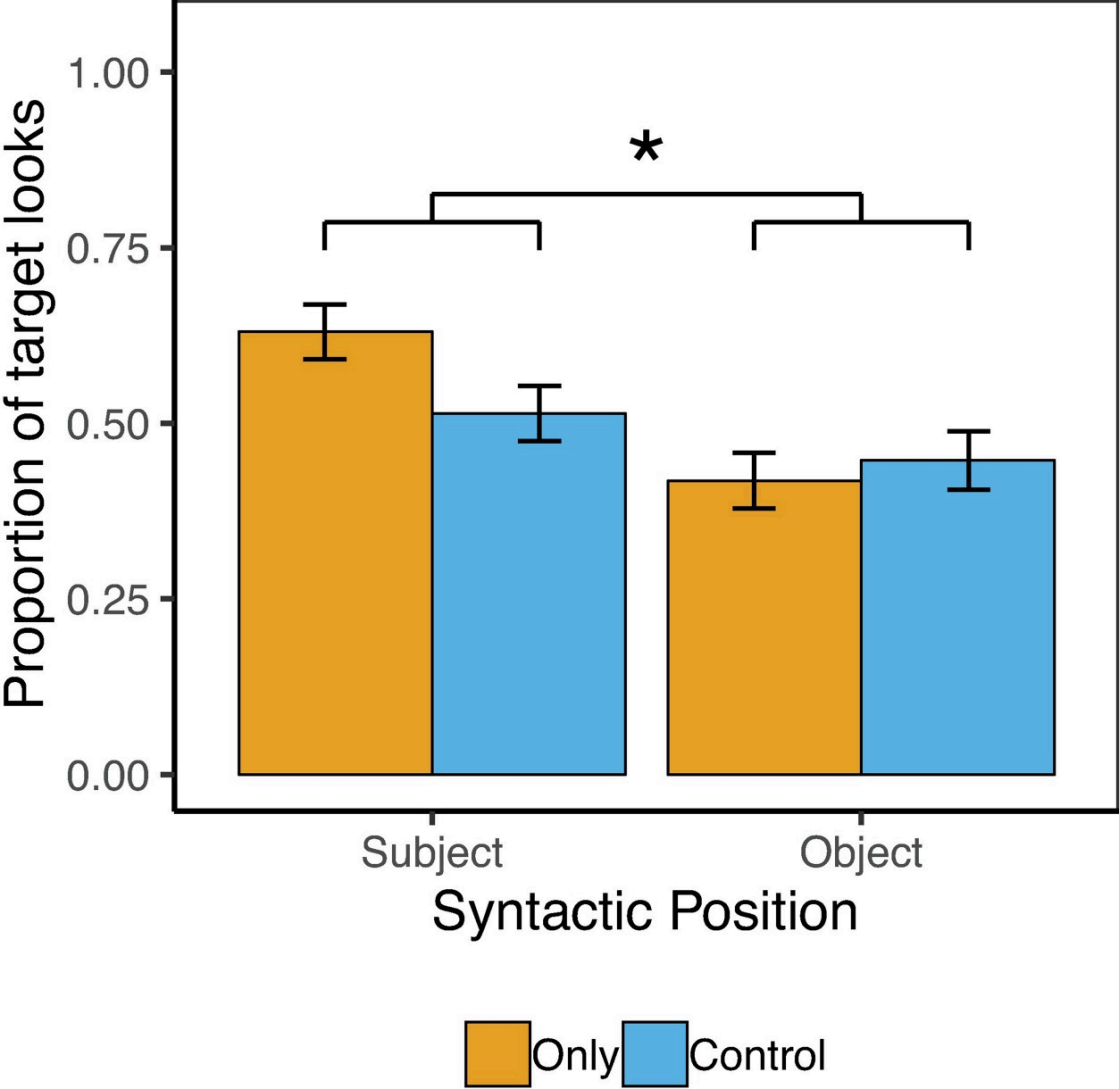
Child results from Exp. 3. Error bars reflect by-subject standard errors.

Target accuracy on the behavioral task was perfect (100%), with no differences by condition. Fig 4 shows the pattern of results for Exp. 3. The overall model revealed significant main effects of Syntactic Position and Block Number, such that child participants looked significantly more to the target image in sentences in which *only* was in Subject vs. Object position (57% vs. 44%), *β* = .27(SE = .07), *z* = 3.66, *p* < .001, and significantly more to the target image in the first vs. second block (55% vs. 47%), *β* = .16(SE = .08), *z* = 2.17, *p* = .03, respectively. There was also a significant Condition-by-Syntactic-Position interaction, *β* = .16(SE = .07), *z* = 2.13, *p* = .03, which follow-up analyses revealed was due to a significant increase in looks to the target in the Subject-Only condition compared to the Subject-Control condition (63% vs. 51%), *β* = .25(SE = .10), *z* = 2.42, *p* = .02, but not in the Object-Only condition relative to Object-Control condition (42% vs. 45%), *β* = -.08(SE = .11), *z* = -.72, *p* = .47. The model revealed no other main effects or interactions.

### Discussion

The main finding from Exp. 3 is that, like adults, children do in fact successfully process subject-*only* sentences online, using the presence of *only* in subject position to make predictions about the upcoming content of the sentence. However, they do not show adult-like processing with object-*only* sentences, suggesting they may not yet have developed the previous mention bias shown to be robust in adults’ online processing of object-*only* sentences (e.g., [25, 26]). We return to this issue in the general discussion. However, as the present study was designed specifically to shed light on children’s online performance with subject-*only* sentences in particular, Exp. 4 was restricted to subject-*only* sentences exclusively in order to verify that children’s success with subject-*only* is indeed robust.

## Experiment 4

### Methods

#### Participants

20 native English-speaking 6- to 8-year-old children (15 female, 5 male; mean age = 7;5 [SD = 9 mos.], range = 6;0–8;7) recruited from the greater Boston area participated in the experiment. The children received a toy for their participation, and their guardians received a $5 travel reimbursement.

#### Materials

The materials for Exp. 4 were the same as for the previous experiments, with one notable exception: The Subject blocks for all eight lists were combined and collapsed into four lists for Exp. 4. Critically, Exp. 4 was not parallel to Exp. 2 because the adults in Exp. 2 were exposed to both sentence types (6 Subject-Only, 6 Subject-Control, 6 Object-Only, 6 Object-Control), while the children in Exp. 4 were given subject-*only* sentences alone (12 Subject-Only, 12 Subject-Control).

#### Procedure

The procedure for Exp. 4 was the same as for the previous experiments.

#### Design

We used a within-subjects design with two levels of Condition (Only, Control). Our dependent variable was the same as in Exp. 3. Participants were randomly assigned to one of four counterbalanced lists.

#### Data analysis

Data preparation for Exp. 4 was the same as for the previous experiments, resulting in 20.2% trial loss. We analyzed the data using a logistic mixed-effects model in the lme4 package in R, with Condition (Only, Control) as the only fixed effect. Maximal random effects structure did not significantly improve model fit, *χ*^2^(4) = .58, *p* = .97, so only random intercepts for participant and item (vignette) were included in the final model. The fixed effect was effect coded.

### Results

**Fig 5:**
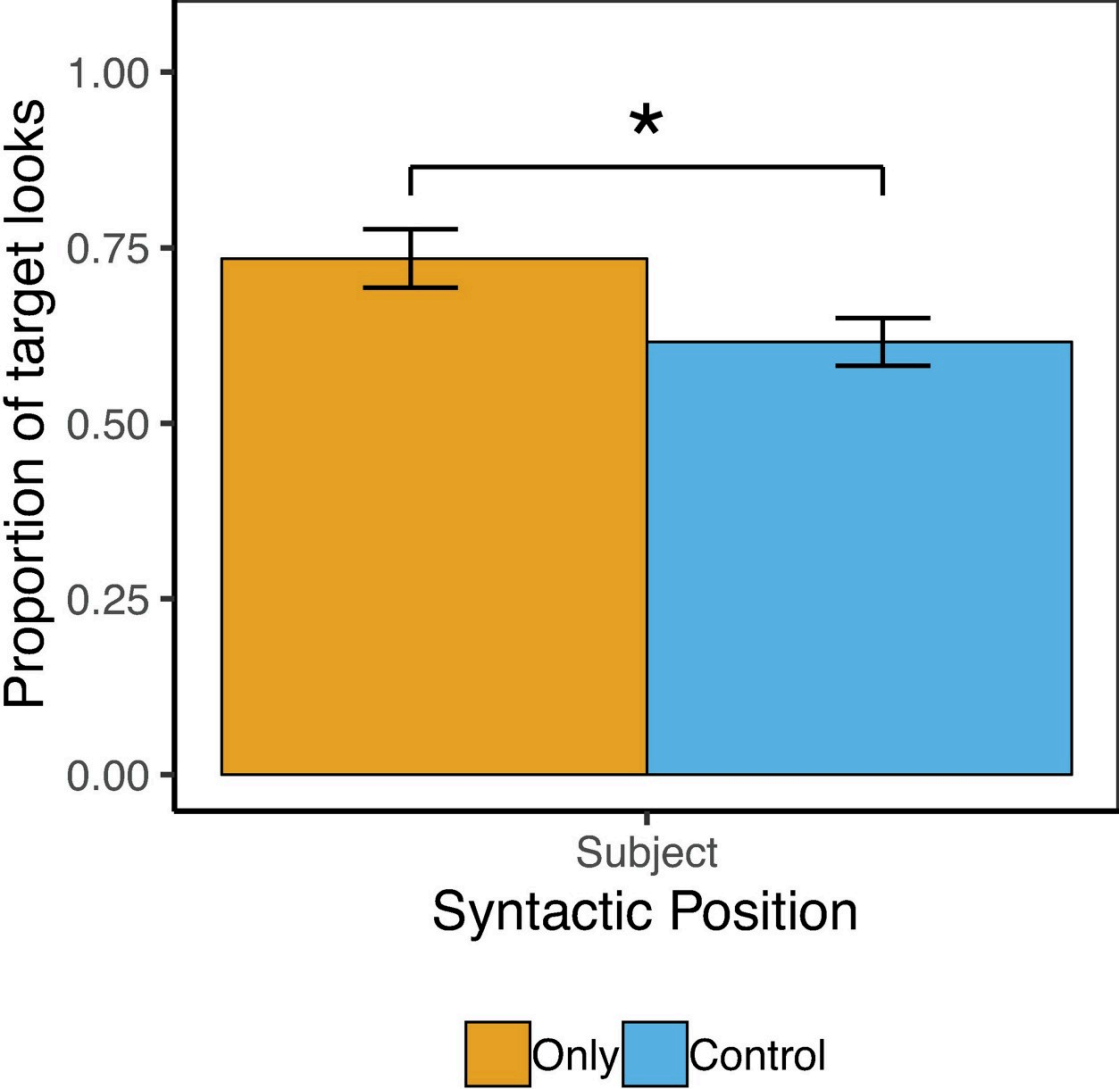
Child results from Exp. 4. Error bars reflect by-subject standard errors.

Target accuracy on the behavioral task was perfect (100%), with no differences by condition. Fig 5 shows the pattern of results for Exp. 4. The overall model revealed a significant main effect of Condition, such that participants looked significantly more to the target image in sentences with Only vs. Control sentences (73% vs. 62%), *β* = .26(SE = .12), *z* = 2.13, *p* = .03.

### Discussion

The results of Exp. 4 replicate and validate our subject-*only* finding from Exp. 3: Children are clearly able to use the presence of subject-*only* to make predictions online about the upcoming content of the sentence, which they used to guide their looking behavior in our task.

## General discussion

This study explored how children interpret sentences with *only* on a moment-to-moment basis in order to better understand the asymmetry between subject- and object-*only* noted in the developmental literature (e.g., [1, 9–16]). We used an online eye-tracking paradigm with a display that was simpler than those in previous adult processing studies (e.g., [23, 25, 26]). Specifically, we reduced the number of alternatives and increased contextual support for the utterances. We found that adults were able to successfully anticipate upcoming referents for both subject- and object-*only* sentences. This is the first study demonstrating that mature listeners can interpret subject-*only* as an utterance unfolds, using it to predict upcoming information in the sentence (cf. [23, 26]). However, we also found interference effects in adults using both a blocked (Exp. 1) and interspersed (Exp. 2) design, suggesting that even adults have difficulty interpreting subject- and object-*only* sentences to the same degree within a single experimental session.

In Exps. 3 and 4, we found that children, like adults, *can* successfully use the presence of subject-*only* to make the prediction that the upcoming referent must be novel. However, our children failed to show an adult-like pattern in the object-*only* condition. Specifically, while previous studies have consistently found a previous mention bias in adults’ online expectations about the target of object-*only* sentences (see [25, 26, 27], our Exp. 1), we found that children as old as 6 to 8 years of age do not yet exhibit this pragmatic bias.

In the remainder of this discussion, we consider the implications of our findings for our understanding of: 1) the prior work on adults’ online interpretation of *only*, and 2) the asymmetry observed in children’s acquisition of *only*, with consequences for the development of language comprehension more generally.

### Implications for processing asymmetry in adults

These studies provide the first evidence that adults are able to use subject-*only* as a cue for predictive processing during online sentence comprehension. But why do they succeed in the current studies when they failed in earlier, similar studies? We suspect this is primarily due to our simplified design, which may have made it easier for participants to make referential predictions for subject-*only* sentences. Computing the subject-*only* inference ultimately requires negating across a set of potential events, as opposed to negating across objects, as in the object-*only* case. As a result, when there are multiple individuals in the contrast set, each must be ruled out sequentially by comparison with the other individuals, making the subject-*only* inference quite complicated. In contrast, computing the object-*only* inference simply requires verifying that the person has only one item in their possession and that that item has already been mentioned. In this paradigm, we had only one other character and thus there was only one individual to model and one other individual to compare to (cf. [23, 26]). As a result, the subject-*only* inference may have been more comparable in complexity to the object-*only* inference in the present study.

Nevertheless, even with this simplified design, these rapid inferences were only made when interference across trials was minimized. Adults in our Exp. 1 (blocked design) successfully predicted the target of *only*-sentences in the first block and failed to do so in the second block, regardless of block order, while adults in Exp. 2 (interspersed design) successfully computed subject-*only* inferences but failed to show the expected previous mention bias with object-*only* sentences (to the point of statistical significance) when the two types of *only-*sentences were interspersed. Why might this be so? Considering a given subject-*only* sentence (*Only Jane ate an apple*) and its minimally different object-*only* counterpart (*Jane only ate an apple*), the explicitly stated proposition remains the same (*Jane ate an apple*). However, the membership of the contrast set changes entirely, leading to markedly different sentence-level interpretations. It could be that switching back and forth between these two different types of inferences for minimally different sentences is cognitively demanding, leading to a slow down in the second block in Exp. 1.

There is also the question of why our adult participants in Exp. 2 performed better with subject-*only* sentences than with object-*only*, in contrast to the poorer performance with subject-*only* reported in past work (cf. [23, 26]). We consider it plausible that performance in our task reflected differences in the reliability of the constraints involved. For example, both sentence types are subject to semantic constraints. In a subject-*only* sentence, it is a semantic requirement that the target item be novel—i.e., not already associated with another character in the contrast set. Similarly, in an object-*only* sentence, it is a semantic requirement that the target be a single item, which both referents in our displays satisfied. Thus, on top of this, our design also hinged on participants exhibiting the well-attested probabilistic bias to expect the previously mentioned target item in object-*only* sentences [25, 26, 27]. Our evidence demonstrates for the first time that adult participants can indeed make both kinds of predictions online, whether *only* associates with the object argument or with the subject argument in the sentence. However, it may very well be that when these contrasting constraints are pitted against each other, as they were in our Exp. 2, participants focused on the more reliable of the two, and here it stands to reason that a hard semantic constraint would trump a more probabilistic bias in this regard. In the prior study by Paul et al. [26] directly comparing the online processing of subject-*only* and object-*only* sentences in adults, where participants failed to make the subject-*only* inference online (likely due to task-specific demands), no such conflict arose, making the object-*only* inference viable.

A related possibility is that our results reflect differences in *absolute* processing time rather than an inability to track both inferences within the same experimental session. In isolation, adult participants are capable of computing both subject- and object-*only* inferences. But it’s possible that the mixing of these two types of *only*-sentences within the same experimental session causes an overall slowdown in participants’ ability to make the relevant predictions. Since *only* occurs at the beginning of subject-*only* sentences, there is a longer time window between it and the critical noun, giving participants more time to make the requisite prediction online. In contrast, in object-*only* sentences, *only* occurs closer to the target noun, giving participants less time to do so. This could explain why we fail to see robust evidence of online target prediction in the object-*only* condition in Exp. 2. However, this explanation cannot account for the opposite pattern of results observed in Paul et al. [26], in which participants reliably showed the expected pattern with object-*only* but not subject-*only* sentences in the same experimental session. Thus, this is unlikely to be the best explanation for our adult data in Exp. 2.

### Implications for acquisition asymmetry in children

Although several previous studies have found that children over age 6 perform above chance on offline comprehension measures of subject-*only*, even in these cases performance on object-*only* was better than for subject-*only* (e.g., [2, 5, 11, 19]). Our results (Exps. 3 and 4) suggest that under the right conditions, children as young as 6 years of age can not only successfully interpret subject-*only* sentences, but they can also do so rapidly online during language comprehension in a manner comparable to our adult sample. We reduced task demands compared to previous offline child studies as well as online adult processing studies in two key ways. First, we used a considerably simplified visual display that made the subject-*only* inference easier to compute in comparison to comparable past studies using the visual-world paradigm [23, 26]. Second, by using an implicit online measure of comprehension, we effectively eliminated the metalinguistic component inherent to explicit offline measures, which is known to be a challenge for young children (see, e.g., [22]). We found that 6- to 8-year-old children were able to use subject-*only* online to anticipate the correct (i.e., semantically congruent) referent.

Importantly, children’s successful use of subject-*only* as a cue for predictive processing is inconsistent with the account put forth by Müller et al. [11, 16] (see also [4]) that children’s difficulty with subject-*only* results from a conflict between the default information structure and that underlying subject-*only* sentences. If issues with representing the requisite focus structure were the root cause of children’s difficulty with subject-*only* sentences in offline tasks, we should see the same pattern arise in online tasks. Specifically, the children in our task should have had difficulty overriding the information structural default, and as such shouldn’t have been able to build the relevant set of alternatives as determined by the presence of subject-*only* in conjunction with context-specific information. Our evidence for children’s anticipatory looks to the target referent of subject-*only* sentences prior to phonological disambiguation shows, to our knowledge for the first time, that children as young as six years of age are able to reliably use syntactic position as a cue to retrieve the underlying focus structure of *only-*sentences. Crucially, this evidence is inconsistent with recent claims that young children ignore the syntactic position of *only* as a cue to focus structure [1, 5].

That children are able to successfully use the syntactic position of *only* as a cue for focus assignment is also interesting in light of past work showing that children have difficulty comprehending prosodic marking of contrastive focus through age 10 [36, 37] (though cf. [6]). In contrast, the 6- to 8-year-olds in our study were able to recover the correct focus structure of subject-*only* sentences using the *syntactic* position of *only* as a cue. For one, this suggests that the comprehension problems observed in the prosody literature need not result from children’s difficulty with representing particular focus structures per se, but instead from differences in the relative accessibility of different types of cues used to mark focus (e.g., prosodic vs. syntactic).

Nevertheless, we did find some aspects of children’s comprehension of *only*-sentences that were not adult-like. Specifically, children in Exps. 3 and 4 consistently failed to show predictive looking to the previously mentioned cohort item in response to the presence of object-*only*. This failure to show an adult-like pattern of looks during the online comprehension of object-*only* sentences cannot be attributed to interference from subject-*only* sentences, as children failed to show a previous mention bias with object-*only* sentences even in the first block of Exp. 3—i.e., where there could have been no such interference. Instead, we suspect that our children may not yet have developed the previous mention bias that leads to predictive processing for adults in the object-*only* case (above and beyond the semantic constraint that the target refer only to a single item, which both referents in our displays satisfied). If this conjecture is correct, we would expect that they would still perform well on measures that do not rely on this bias, such as offline measures of comprehension. It could be that the previous mention bias emerges after considerably more exposure, which children may not receive enough of until past 8 years of age, the age of the oldest children in our study. One way to address this possibility would be to examine the frequency of object-*only* sentences in corpora of child-directed speech and the strength of the previous mention bias in these sentences.

An alternative explanation, discussed above, is that our child participants simply had more time to compute the subject-*only* inference than they had to compute the object-*only* inference. In subject-*only* sentences, *only* occurs at the beginning of the sentence, whereas in object-*only* sentences it occurs closer to the target noun. In principle, this might have given them more time to predict the intended referent for subject-*only* sentences relative to object-*only* sentences. This interpretation doesn’t fit with the adult literature, however: Two recent studies failed to find evidence for incremental processing of subject-*only* sentences in adults [23, 26], while success with object-*only* sentences has been found and replicated multiple times [25, 26], including in the present paper. Moreover, there is ample evidence for rapid prediction of this sort by children of our age range (i.e., 6- to 8-year-olds) and even younger [6, 38, 39]. Thus, this is unlikely to be the explanation for our findings.

Despite children’s non-adult-like performance in the object-*only* condition of our task, the overall pattern nevertheless shows that they *are* able to integrate linguistic information across many distinct levels of representation incrementally during language comprehension. To correctly anticipate the target of *only*-sentences in our study, our child and adult participants needed to integrate the lexical semantics of *only*, its syntactic position, and, crucially, the discourse and visual contexts within which these sentences appeared. Specifically, predictive looks in our subject-*only* conditions required the ability to integrate contextually-given information with the information provided by the sentence in order to uniquely identify the novel item picked by the target character. That children are able to do this rapidly online before the sentence has ended suggests that this process occurs simultaneously with the construction of the semantic and syntactic structures of these sentences, rather than proceeding secondarily to linguistic composition. This is hard to reconcile with theoretical models of language which posit that contextual processes are strictly relegated to a post-compositional stage (e.g., [8]).

## Conclusion

We have found evidence that both children and adults can successfully process subject-*only* sentences online. However, 6- to 8-year-old children showed a non-adult-like pattern with object-*only* sentences, suggesting that they have yet to develop the previous mention bias that drives predictions with this sentence type in adults. We also found that while adults are able to rapidly make predictions for both types of *only*-sentences online, they have difficulty switching back and forth between subject- and object-*only* sentences in the same experimental session, suggesting a possible interference effect. As a whole, these findings suggest that much of the asymmetry in performance for subject- vs. object-*only* sentences noted in past work was due to task demands. Under the right conditions, children and adults are able to predictively process sentences where *only* modifies the subject argument. Finally, these findings provide further evidence for the rapid integration of multiple sources of linguistic and non-linguistic information in real time during children’s and adults’ sentence processing, suggesting that contextual factors can and do actively feed into compositional processes, rather than being integrated post-compositionally as expected under a strictly modular view.

## Supporting information

S1 Table Critical stimuli. (DOCX)
