# Supplementary material for: Children and adults successfully comprehend subject-only sentences online
Source: PLoS One. 2019 Jan 17;14(1):e0209670. doi: 10.1371/journal.pone.0209670 (PMC6336383; doi:10.1371/journal.pone.0209670)
Supplement: S1 Table — (DOCX) [file pone.0209670.s001.docx]

**S1 Table. Critical stimuli.**

| **Item** | **Discourse context** | **Subject-*only*** | **Object-*only*** | **Controls** |
| --- | --- | --- | --- | --- |
| 1 | Buzz Lightyear and friends went on a trip to the farmer's market.  Buzz Lightyear picked the apple/apricot and the pear. | Only Woody picked the apricot/apple. | Woody only picked the apple/apricot. | And Woody picked the apple/apricot. |
| 2 | Buzz Lightyear and friends went on a trip to the zoo.  Buzz Lightyear picked the panda/parrot and the rhinoceros. | Only Woody picked the parrot/panda. | Woody only picked the panda/parrot. | And Woody picked the panda/parrot. |
| 3 | Buzz Lightyear and friends went on a trip to the carnival.  Buzz Lightyear picked the popsicle/popcorn and the hotdog. | Only Woody picked the popcorn/popsicle. | Woody only picked the popsicle/popcorn. | And Woody picked the popsicle/popcorn. |
| 4 | Buzz Lightyear and friends went on a trip to the aquarium.  Buzz Lightyear picked the seashell/seagull and the turtle. | Only Woody picked the seagull/seashell. | Woody only picked the seashell/seagull. | And Woody picked the seashell/seagull. |
| 5 | Winnie the Pooh and friends went to school.  Winnie the Pooh picked the chalk/chocolate milk and the water bottle. | Only Tigger picked the chocolate milk/chalk. | Tigger only picked the chalk/chocolate milk. | And Tigger picked the chalk/chocolate milk. |
| 6 | Winnie the Pooh and friends went to a birthday party.  Winnie the Pooh picked the candle/candy and the balloon. | Only Tigger picked the candy/candle. | Tigger only picked the candle/candy. | And Tigger picked the candle/candy. |
| 7 | Winnie the Pooh and friends went on a trip to a farm.  Winnie the Pooh picked the cabbage/carrot and the scarecrow. | Only Tigger picked the carrot/cabbage. | Tigger only picked the cabbage/carrot. | And Tigger picked the cabbage/carrot. |
| 8 | Winnie the Pooh and friends went outside to the playground.  Winnie the Pooh picked the skateboard/skates and the scooter. | Only Tigger picked the skates/skateboard. | Tigger only picked the skateboard/skates. | And Tigger picked the skateboard/skates. |
| 9 | Big Bird and friends went on a trip to the mall.  Big Bird picked the wallet/watch and the scarf. | Only Elmo picked the watch/wallet. | Elmo only picked the wallet/watch. | And Elmo picked the wallet/watch. |
| 10 | Big Bird and friends went on a trip to the toy store.  Big Bird picked the teddy bear/telescope and the jigsaw puzzle. | Only Elmo picked the telescope/teddy bear. | Elmo only picked the teddy bear/telescope. | And Elmo picked the teddy bear/telescope. |
| 11 | Big Bird and friends went on a trip to the beach.  Big Bird picked the sandcastle/sandals and the frisbee. | Only Elmo picked the sandals/sandcastle. | Elmo only picked the sandcastle/sandals. | And Elmo picked the sandcastle/sandals. |
| 12 | Big Bird and friends went on a trip to the candy store.  Big Bird picked the candy cane/candy corn and the jellybeans. | Only Elmo picked the candy corn/candy cane. | Elmo only picked the candy cane/candy corn. | And Elmo picked the candy cane/candy corn. |
| 13 | Big Bird and friends went on a trip to the farmer's market.  Big Bird picked the grapes/grapefruit and the banana. | Only Elmo picked the grapefruit/grapes. | Elmo only picked the grapes/grapefruit. | And Elmo picked the grapes/grapefruit. |
| 14 | Big Bird and friends went on a trip to a farm.  Big Bird picked the cowboy hat/cow and the rooster. | Only Elmo picked the cow/cowboy hat. | Elmo only picked the cowboy hat/cow. | And Elmo picked the cowboy hat/cow. |
| 15 | Big Bird and friends went on a trip to the aquarium.  Big Bird picked the seahorse/sea lion and the jellyfish. | Only Elmo picked the sea lion/seahorse. | Elmo only picked the seahorse/sea lion. | And Elmo picked the seahorse/sea lion. |
| 16 | Big Bird and friends went outside to the playground.  Big Bird picked the basketball/backpack and the jump rope. | Only Elmo picked the backpack/basketball. | Elmo only picked the basketball/backpack. | And Elmo picked the basketball/backpack. |
| 17 | Buzz Lightyear and friends went to a birthday party.  Buzz Lightyear picked the present/pretzel and the streamers. | Only Woody picked the pretzel/present. | Woody only picked the present/pretzel. | And Woody picked the present/pretzel. |
| 18 | Buzz Lightyear and friends went on a trip to the beach.  Buzz Lightyear picked the sunscreen/sunglasses and the beachball. | Only Woody picked the sunglasses/sunscreen. | Woody only picked the sunscreen/sunglasses. | And Woody picked the sunscreen/sunglasses. |
| 19 | Buzz Lightyear and friends went on a trip to the toy store.  Buzz Lightyear picked the dollhouse/dolphin and the jack-in-the-box. | Only Woody picked the dolphin/dollhouse. | Woody only picked the dollhouse/dolphin. | And Woody picked the dollhouse/dolphin. |
| 20 | Buzz Lightyear and friends went to school.  Buzz Lightyear picked the marbles/markers and the lunchbox. | Only Woody picked the markers/marbles. | Woody only picked the marbles/markers. | And Woody picked the marbles/markers. |
| 21 | Winnie the Pooh and friends went on a trip to the carnival.  Winnie the Pooh picked the ice cream/ice cube and the cotton candy. | Only Tigger picked the ice cube/ice cream. | Tigger only picked the ice cream/ice cube. | And Tigger picked the ice cream/ice cube. |
| 22 | Winnie the Pooh and friends went on a trip to the zoo.  Winnie the Pooh picked the polar bear/pony and the flamingo. | Only Tigger picked the pony/polar bear. | Tigger only picked the polar bear/pony. | And Tigger picked the polar bear/pony. |
| 23 | Winnie the Pooh and friends went on a trip to the mall.  Winnie the Pooh picked the earmuffs/earrings and the sweater. | Only Tigger picked the earrings/earmuffs. | Tigger only picked the earmuffs/earrings. | And Tigger picked the earmuffs/earrings. |
| 24 | Winnie the Pooh and friends went on a trip to the candy store.  Winnie the Pooh picked the gum balls/gummy bears and the licorice. | Only Tigger picked the gummy bears/gum balls. | Tigger only picked the gum balls/gummy bears. | And Tigger picked the gum balls/gummy bears. |
